# Prognostic role of pretreatment skeletal muscle index in gastric cancer patients: A meta-analysis

**DOI:** 10.3389/pore.2023.1611055

**Published:** 2023-04-24

**Authors:** Xiaohong He, Sicheng Zhou, Hongjun Li, Yue Gou, Dan Jia

**Affiliations:** ^1^ Cancer Center, West China Hospital, Sichuan University, Chengdu, China; ^2^ Outpatient Department, West China Hospital, Sichuan University/West China School of Nusing Sichuan University, Chengdu, China

**Keywords:** meta-analysis, prognosis, gastric cancer, pretreatment, skeletal muscle index

## Abstract

**Background:** The association between pretreatment skeletal muscle index (SMI) and long-term survival of gastric cancer patients remains unclear up to now. The aim of this meta-analysis was to identify the prognostic value of pretreatment SMI in gastric cancer.

**Methods:** The PubMed, EMBASE and Web of Science electronic databases were searched up to 5 June 2022 for relevant studies. The primary outcome was overall survival (OS) and the second outcomes were disease-free survival (DFS) and cancer-specific survival (CSS). The hazard ratios (HRs) and 95% confidence intervals (CIs) were combined to assess the relationship between pretreatment SMI and survival of gastric cancer patients. All statistical analyses were conducted by STATA 15.0 software.

**Results:** A total of 31 retrospective studies involving 12,434 patients were enrolled in this meta-analysis. The pooled results demonstrated that lower pretreatment was significantly associated with poorer OS (HR = 1.53, *p* < 0.001). Besides, lower pretreatment SMI was also related with worse DFS (HR = 1.39, *p* < 0.001) and CSS (HR = 1.96, *p* < 0.001).

**Conclusion:** Pretreatment SMI was significantly associated with prognosis of gastric cancer patients and lower SMI predicted worse survival. However, more prospective high-quality studies are still needed to verify our findings.

## Introduction

Gastric cancer remains the sixth highest incidence of all cancer worldwide with over none million newly diagnosed cases every year and is also the second-leading cause of cancer-related death (1). Despite of the great advances in the early diagnosis and treatment strategies including the screening of gastroscopy, surgical techniques and neoadjuvant chemoradiotherapy, the prognosis of gastric cancer patients remains extremely poor and about half of gastric cancer patients may recur after curative treatment, which is a severe global health problem needing to be solved (2, 3). It is still necessary to identify more valuable and reliable clinical indicators to precisely estimate the prognosis of gastric cancer patients.

It is well known that the nutrition condition of the body plays an essential role in the development and progression of cancers. In overall, a poor nutrition condition could promote the occurrence and progression of cancers and malnutrition leads to a decline in the antineoplastic ability of patients (4, 5). Besides, the nutritional status could help predict long-term survival of cancer patients (4, 5). In recent years, a number of clinical parameters which could reflect the nutritional status to a certain extent have been introduced and reported to show high prognostic value in gastric cancer such as the controlling nutritional status (CONUT) score, prognostic nutritional index (PNI) and geriatric nutritional risk index (GNRI) (6–8). Unfortunately, none of these parameters are widely applied in clinics because they are unstable and could be affected by many factors.

Skeletal muscle loss is a strong and reliable indicator for poor nutritional status and closely related with survival of cancer patients (9–11). In recent years, skeletal muscle index has been introduced and it is calculated based on the third lumbar vertebra (L3) level skeletal muscle area and squared height (12). Meanwhile, the skeletal muscles in the L3 level include the e psoas muscles, the para-spinal muscles, and the abdominal wall muscles. Furthermore, the prognostic value of pretreatment SMI in several cancers has been well verified such as the epithelial ovarian cancer, colorectal cancer and hepatocellular carcinoma (13–15). However, the prognostic role of pretreatment SMI in gastric cancer remains unclear.

Thus, the aim of this meta-analysis was to demonstrate the prognostic value of pretreatment SMI in gastric cancer, which might contribute to predicting and improving the clinical outcomes of gastric cancer patients.

## Materials and methods

This systematic review and meta-analysis were conducted according to the Preferred Reporting Items for Systematic Reviews and Meta-Analyses guidelines (16).

### Literature search

The PubMed, EMBASE and Web of Science electronic databases were searched up to 5 June 2022. The following key words were used during the search: skeletal muscle index, SMI, stomach, gastric, neoplasm, cancer, tumor, carcinoma, survival, prognosis and prognostic. A combination of subject terms and free words was applied. The detailed search strategy was as follows: (skeletal muscle index OR SMI) AND (stomach OR gastric) AND (cancer OR tumor OR neoplasm OR carcinoma) AND (prognosis OR survival OR prognostic). Besides, the references cited in included studies were also reviewed for eligibility.

### Inclusion and exclusion criteria

The inclusion criteria were as follows: 1) patients were pathologically diagnosed with primary gastric cancer; 2) the SMI was calculated before any anti-tumor treatment such as the surgery, chemotherapy and radiotherapy; 3) the association between pretreatment SMI and survival of gastric cancer patients were explored and the corresponding hazard ratios (HRs) and 95% confidence intervals (CIs) were reported in articles directly; 4) the outcomes included at least one of the following endpoints: overall survival (OS), disease-free survival (DFS) and cancer-specific survival (CSS).

The exclusion criteria were as follows: 1) meeting abstracts, animal trials, case reports, reviews or editorials; 2) HRs with corresponding 95% CIs were unavailable; 3) full texts were not available; 4) low-quality studies with the Newcastle-Ottawa Scale (NOS) score of 5 or lower (17).

### Data collection and quality assessment

In this meta-analysis, the following information was collected from included studies: the name of first author, publication year, sample size, country, threshold of SMI, tumor-node-metastasis (TNM) stage, treatment, endpoint, HR and corresponding 95% CI.

The NOS was applied to evaluate the quality of included studies and only high-quality studies with a NOS score of 6 or higher were included (17).

The literature search, selection, data collection and quality assessment were all performed by two authors independently.

### Statistical analysis

All statistical analyses were conducted with STATA 15.0 software. The HRs with corresponding 95% CIs were calculated to evaluate the association between pretreatment SMI and prognosis of gastric cancer patients. The heterogeneity among the included studies was evaluated by I^2^ statistics and Q test. When significant heterogeneity was observed representing as I^2^ > 50% and (or) *p* < 0.1, the random effects model was applied; otherwise, the fix effects model was used. The sensitivity analysis was performed to detect the sources of heterogeneity and evaluate the stability of pooled results. Furthermore, the Begg’s funnel plot and Egger’s test were conducted to detect publication bias and significant publication bias was defined as *p* < 0.05 (18, 19) If significant publication bias was detected, then the nonparametric trim-and-fill method was applied to re-estimate a corrective effect size after publication bias was adjusted (20).

## Results

### Literature search

**FIGURE 1:**
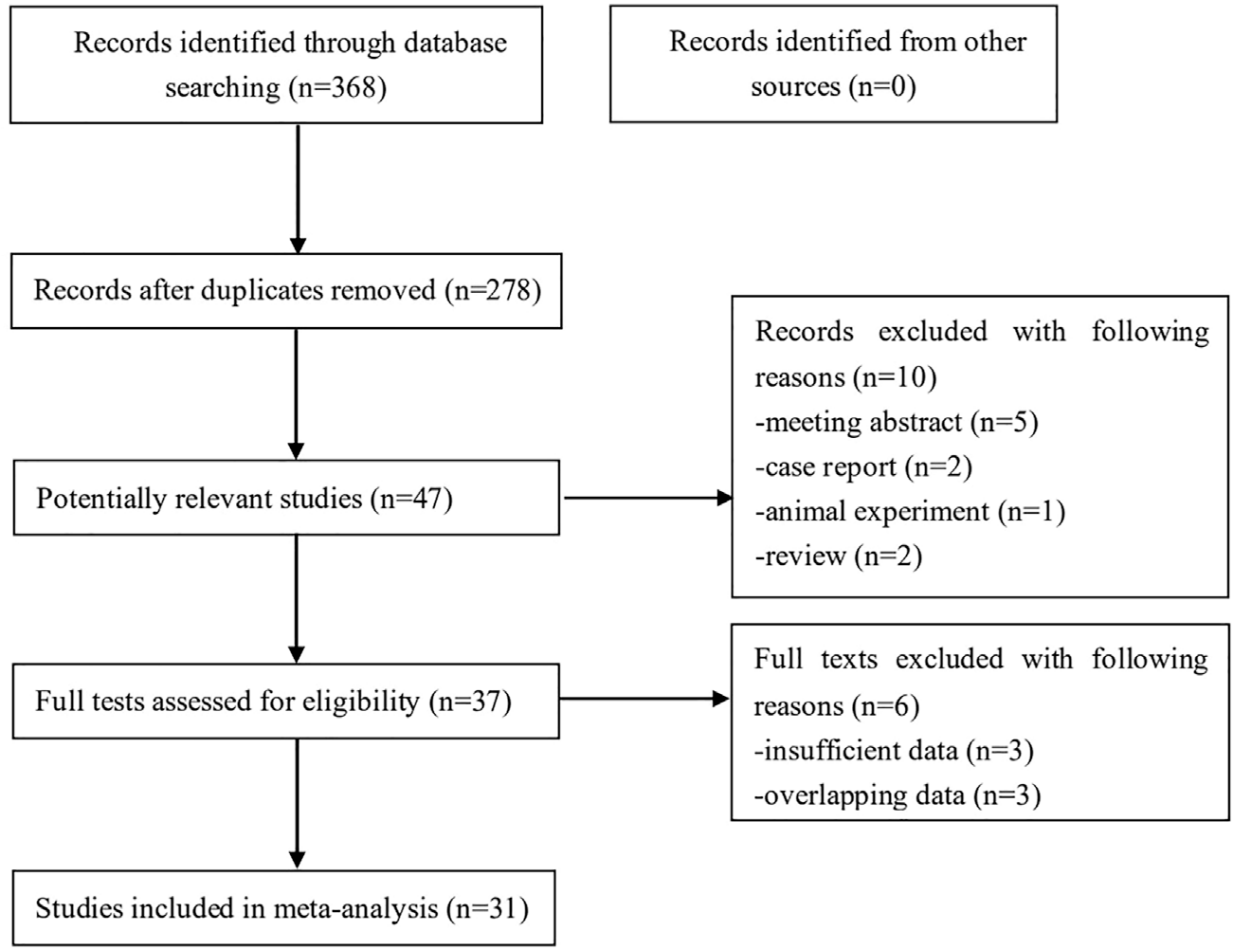
The flow diagram of this meta-analysis.

Initially, 368 records were identified from several databases and 90 duplicated records were removed. Then 47 potentially relevant publications were further reviewed and ten records were excluded. Eventually, 29 articles were included (21–49). However, in the studies conducted by (29) and Wang et al. (32), two different populations were enrolled. Thus, we considered them as two studies separately and a total of 31 studies were included. The detailed literature search process was presented in Figure 1.

### Basic characteristics of included studies

Among these 31 studies, 12,434 patients were enrolled and the sample size ranged from 40 to 1801. These studies were reported from 2016 to 2022. Most of patients were from Asian countries (26/31) and received surgical treatment (25/31). All included studies were high-quality studies with a NOS score ≥6. The other detailed characteristics were presented in Table 1.

**TABLE 1:** Basic characteristics of included studies.

Author	Year	Sample size	Country	Threshold of SMI	TNM	Treatment	Endpoint	NOS
Hayashi (21)	2016	53	Japan	41 cm^2^/m^2^ for women, 43/53 cm^2^/m^2^ for men	Advanced	Non-surgery	OS	6
Zhuang (22)	2016	937	China	34.9cm2/m2 for women, 40.8cm2/m2 for men	I-III	Surgery	OS, DFS	7
Zheng (23)	2017	924	China	28.6 cm^2^/m^2^ for women, 32.5 cm^2^/m^2^ for men	NR	Surgery	OS, DFS	7
Lee (24)	2018	140	South Korea	31 cm^2^/m^2^ for women, 49 cm^2^/m^2^ for men	Advanced	Non-surgery	OS	7
O’Brien (25)	2018	56	Ireland	38.5 cm^2^/m^2^ for women, 52.4 cm^2^/m^2^ for men	I-III	Surgery	OS	8
Park (26)	2018	136	Korea	49.4 cm^2^/m^2^	II-III	Surgery	OS	7
Sugiyama (27)	2018	231	Japan	38.5 cm^2^/m^2^ for women, 53.4 cm^2^/m^2^ for men	IV	Non-surgery	OS	6
Sierzega (28)	2019	138	Poland	38.5 cm^2^/m^2^ for women, 52.4 cm^2^/m^2^ for men	I-IV	Surgery	OS	7
Zhuang (29)	2019	150	China	34.9 cm^2^/m^2^ for women, 40.8 cm^2^/m^2^ for men	I-III	Surgery	OS, DFS	7
Zhuang (29)	2019	167	China	34.9 cm^2^/m^2^ for women, 40.8 cm^2^/m^2^ for men	I-III	Surgery	OS, DFS	7
Kim (30)	2020	305	Korea	53.6 cm^2^/m^2^ for women, 56.2 cm^2^/m^2^ for men	I-III	Surgery	OS	6
Il Yu (31)	2020	440	Republic of Korea	31 cm^2^/m^2^ for women, 49 cm^2^/m^2^ for men	NR	Surgery	OS, DFS	6
Dong (32)	2020	1,147	China	34.9 cm^2^/m^2^ for women, 40.8 cm^2^/m^2^ for men	I-III	Surgery	OS, DFS	6
Yang (33)	2020	182	China	38.5 cm^2^/m^2^ for women, 52.4 cm^2^/m^2^ for men	NR	Surgery	OS	7
Wang (34)	2020	64	China	32.4 cm^2^/m^2^ for women, 44.3 cm^2^/m^2^ for men	NR	Surgery	OS, DFS	6
Wang (34)	2020	74	China	32.4 cm^2^/m^2^ for women, 44.3 cm^2^/m^2^ for men	NR	Surgery	OS, DFS	6
An (35)	2021	339	Republic of Korea	40.77 cm^2^/m^2^ for women, 46.48 cm^2^/m^2^ for men	I-III	Surgery	OS, DFS	7
Alnimri (36)	2021	62	Australia	41 cm^2^/m^2^ for women, 43/53 cm^2^/m^2^ for men	I-IV	Surgery	OS, DFS	7
Huang (37)	2021	597	China	34.9 cm^2^/m^2^ for women, 40.8 cm^2^/m^2^ for men	I-III	Surgery	OS, DFS	6
Kim J (38)	2021	840	South Korea	31 cm^2^/m^2^ for women, 49 cm^2^/m^2^ for men	I-IV	Surgery	OS	7
Kim KW (39)	2021	958	South Korea	None	IIA-IIIC	Surgery	OS	6
Kim YY (40)	2021	149	Korea	31 cm^2^/m^2^ for women, 49 cm^2^/m^2^ for men	Advanced	Non-surgery	OS, PFS	7
Matsunaga (41)	2021	83	Japan	34.7 cm^2^/m^2^ for women, 43.9 cm^2^/m^2^ for men	Advanced	Non-surgery	OS	7
Lee (42)	2021	1801	South Korea	34.1 cm^2^/m^2^ for women, 44.4 cm^2^/m^2^ for men	I-III	Surgery	OS, DFS	7
Rimini (43)	2021	40	Italy	41 cm^2^/m^2^ for women, 43/53 cm^2^/m^2^ for men	IV	Non-surgery	OS	6
Sakurai (44)	2021	1,054	Japan	34.9 cm^2^/m^2^ for women, 40.8 cm^2^/m^2^ for men	I-III	Surgery	OS, CSS	7
Taki (45)	2021	257	Japan	37.3 cm^2^/m^2^ for women, 39.0 cm^2^/m^2^ for men	I-III	Surgery	OS	7
Matsui (46)	2022	512	Japan	34.04 cm^2^/m^2^ for women, 41.87 cm^2^/m^2^ for men	I-IV	Surgery	OS	7
Ricciardolo (47)	2022	55	Italy	38.5 cm^2^/m^2^ for women, 52.4 cm^2^/m^2^ for men	I-IV	Surgery	OS, DFS	7
Tan (48)	2022	318	China	37.81 cm^2^/m^2^ for women, 43.13 cm^2^/m^2^ for men	I-III	Surgery	OS, DFS	7
Xiong (49)	2022	225	China	34.9 cm^2^/m^2^ for women, 40.8 cm^2^/m^2^ for men	I-III	Surgery	OS, DFS	6

NR, not reported; OS, overall survival; DFS, disease-free survival; CSS, cancer-specific survival; NOS, Newcastle-Ottawa Scale.

### The association between pretreatment SMI and OS of gastric cancer patients

**FIGURE 2:**
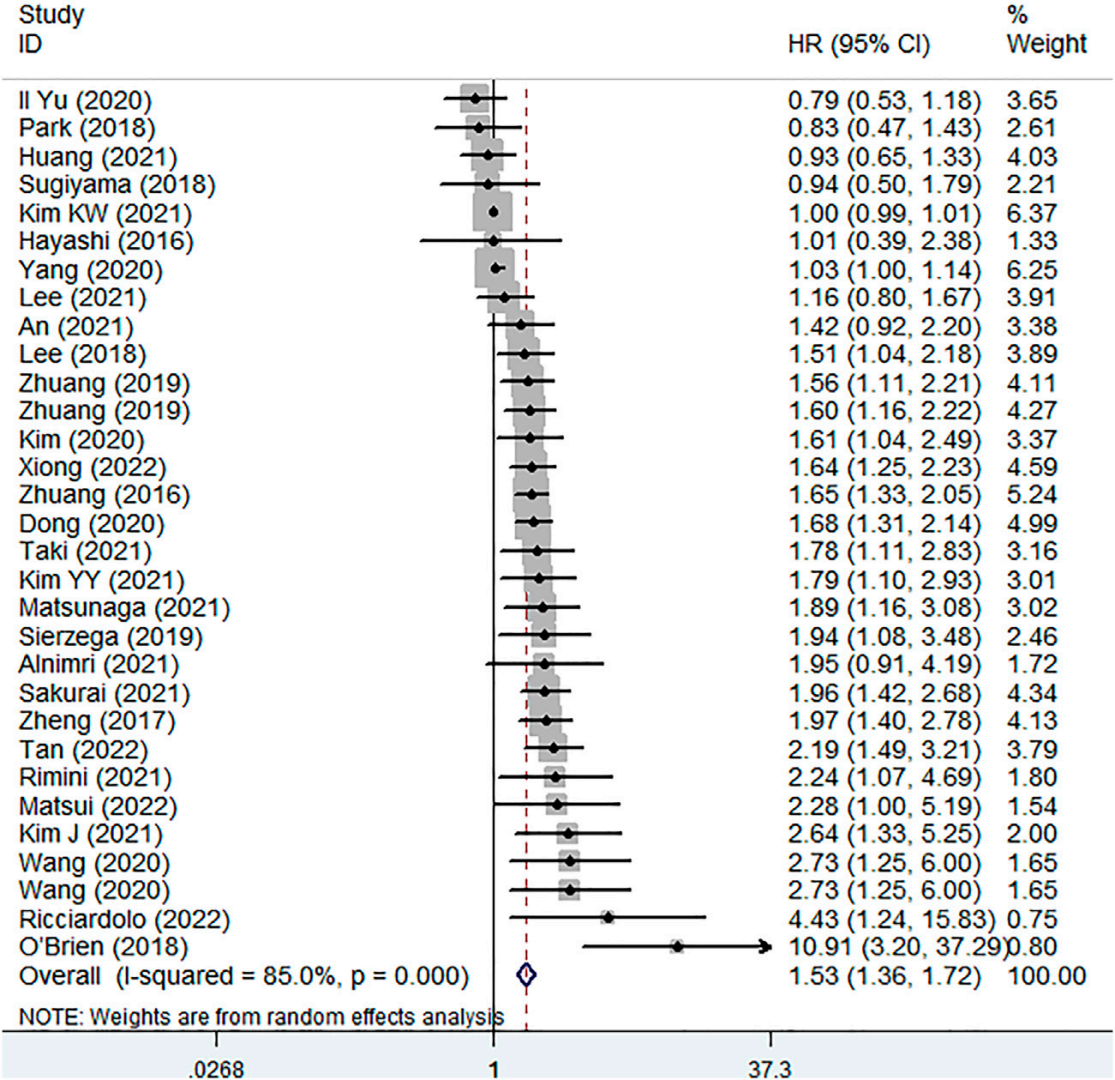
The association between pretreatment skeletal muscle index and overall survival in gastric cancer.

All included studies explored the predictive role of pretreatment SMI for OS of gastric cancer patients (21–49). The pooled results demonstrated that lower pretreatment SMI was significantly associated with poorer OS (HR = 1.53, 95% CI: 1.36–1.72, *p* < 0.001; I^2^ = 85.0%, *p* < 0.001) (Figure 2).

Furthermore, the subgroup analysis based on the country (non-China: HR = 1.56, 95% CI: 1.27–1.91, *p* < 0.001; China: HR = 1.61, 95% CI: 1.29–2.01, *p* < 0.001) and treatment (non-surgery: HR = 1.56, 95% CI: 1.25–1.95, *p* < 0.001; surgery: HR = 1.53, 95% CI: 1.35–1.73, *p* < 0.001) were also conducted and similar results were observed (Table 2).

**TABLE 2:** Results of meta-analysis.

	No. of studies	HR	95% CI	*p*-value	I^2^ (%)	*p*-value
Overall survival	31	1.53	1.36–1.72	<0.001	85.0	<0.001
Country						
Non-China	20	1.56	1.27–1.91	<0.001	79.5	<0.001
China	11	1.61	1.29–2.01	<0.001	86.5	<0.001
Treatment						
Non-surgery	6	1.56	1.25–1.95	<0.001	2.8	0.399
Surgery	25	1.53	1.35–1.73	<0.001	86.6	<0.001
Disease-free survival	13	1.39	1.13–1.69	0.001	58.5	0.004
Cancer-specific survival	1	1.96	1.42–2.68	<0.001	—	—

HR, hazard ratio; CI, confidence interval.

### The association between pretreatment SMI and DFS of gastric cancer patients

**FIGURE 3:**
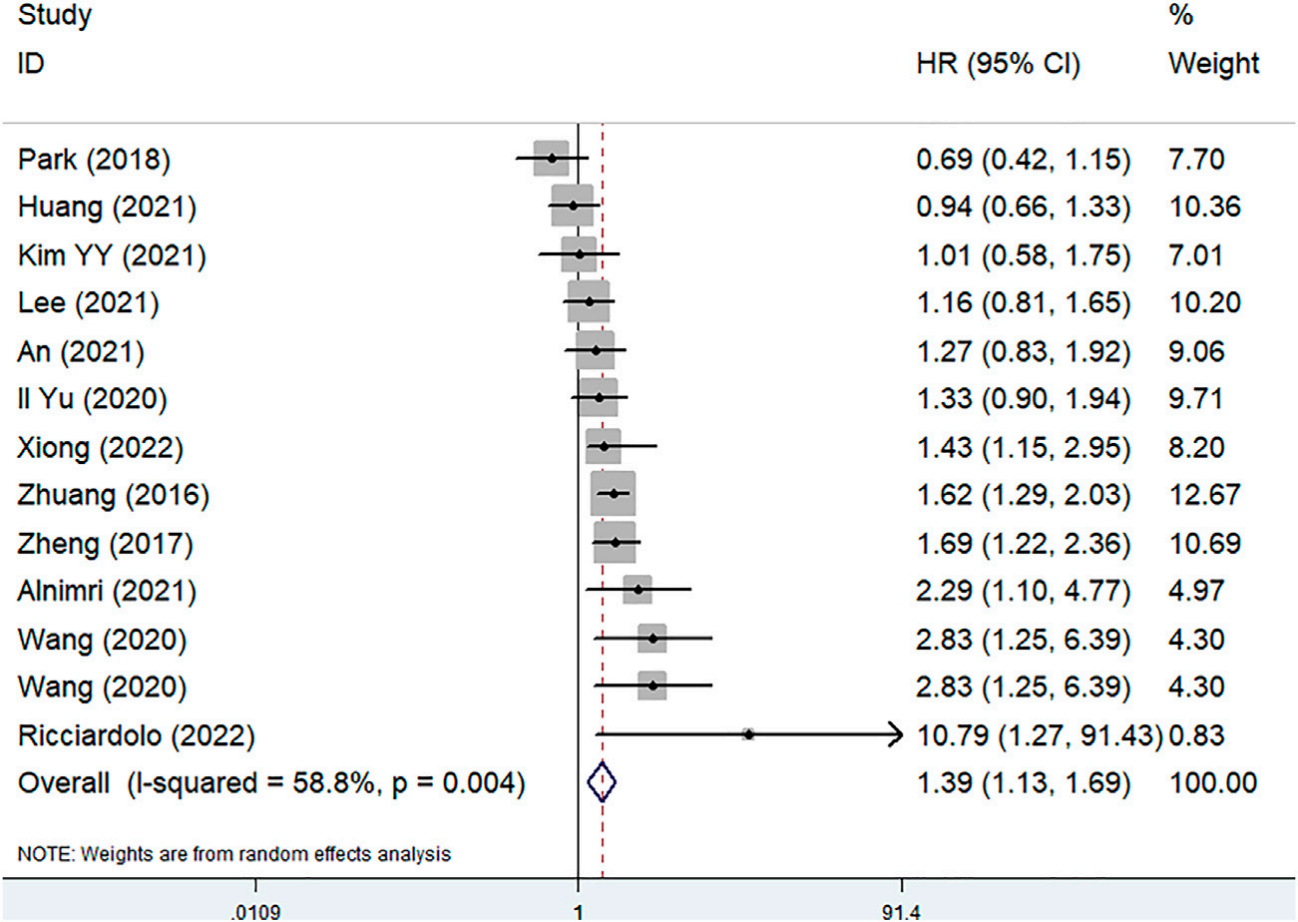
The association between pretreatment skeletal muscle index and disease free survival in gastric cancer.

Only 13 included studies involving 5,803 patients explored the predictive role of pretreatment SMI for DFS (22, 23, 26, 32, 34–37, 40, 41, 47, 49). The pooled results indicated that lower pretreatment SMI was related with worse DFS (HR = 1.39, 95% CI: 1.13–1.69, *p* < 0.001; I^2^ = 58.5%, *p* = 0.004) (Figure 3).

Besides, subgroup analysis based on the country (non-China: HR = 1.22 95% CI: 0.92–1.62, *p* = 0.001; China: HR = 1.57, 95% CI: 1.19–2.06, *p* = 0.176) and treatment (surgery: HR = 1.42, 95% CI: 1.15–1.75, *p* = 0.001) were also performed and similar results were observed (Table 2).

### The association between pretreatment SMI and CSS of gastric cancer patients

Only one study involving 1,054 patients explored the predictive role of pretreatment SMI for CSS (44). The results manifested that patients with a lower pretreatment SMI showed significantly worse CSS (HR = 1.96, 95% CI: 1.42–2.68, *p* < 0.001) (Table 2).

### Sensitivity analysis and publication bias

**FIGURE 4:**
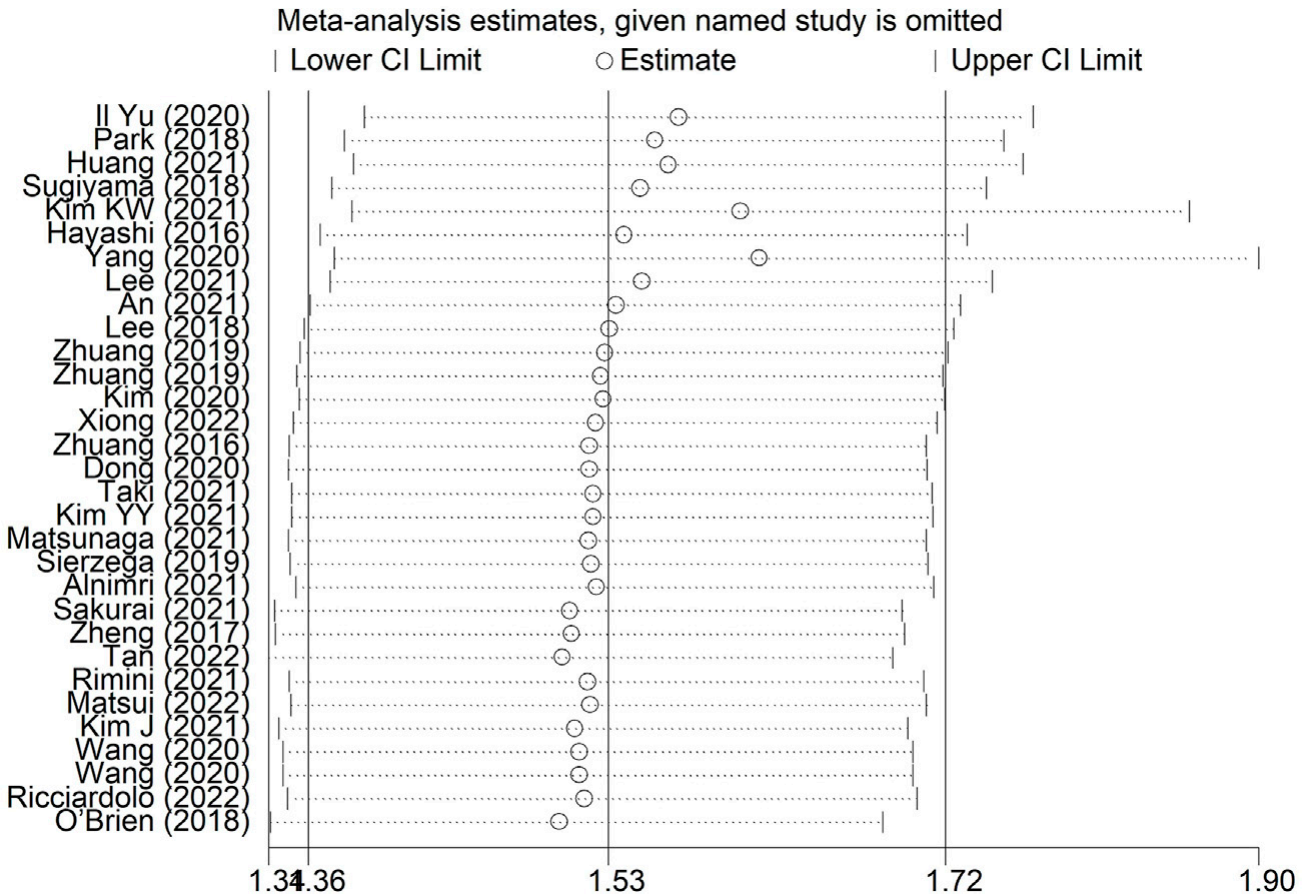
Sensitivity analysis about the association between pretreatment skeletal muscle index and overall survival in gastric cancer.

The sensitivity analysis was conducted by excluding each included studies at one time, which indicated that the pooled results of this meta-analysis were stable and reliable and none of each included studies had a significantly impact on the overall results (Figure 4).

**FIGURE 5:**
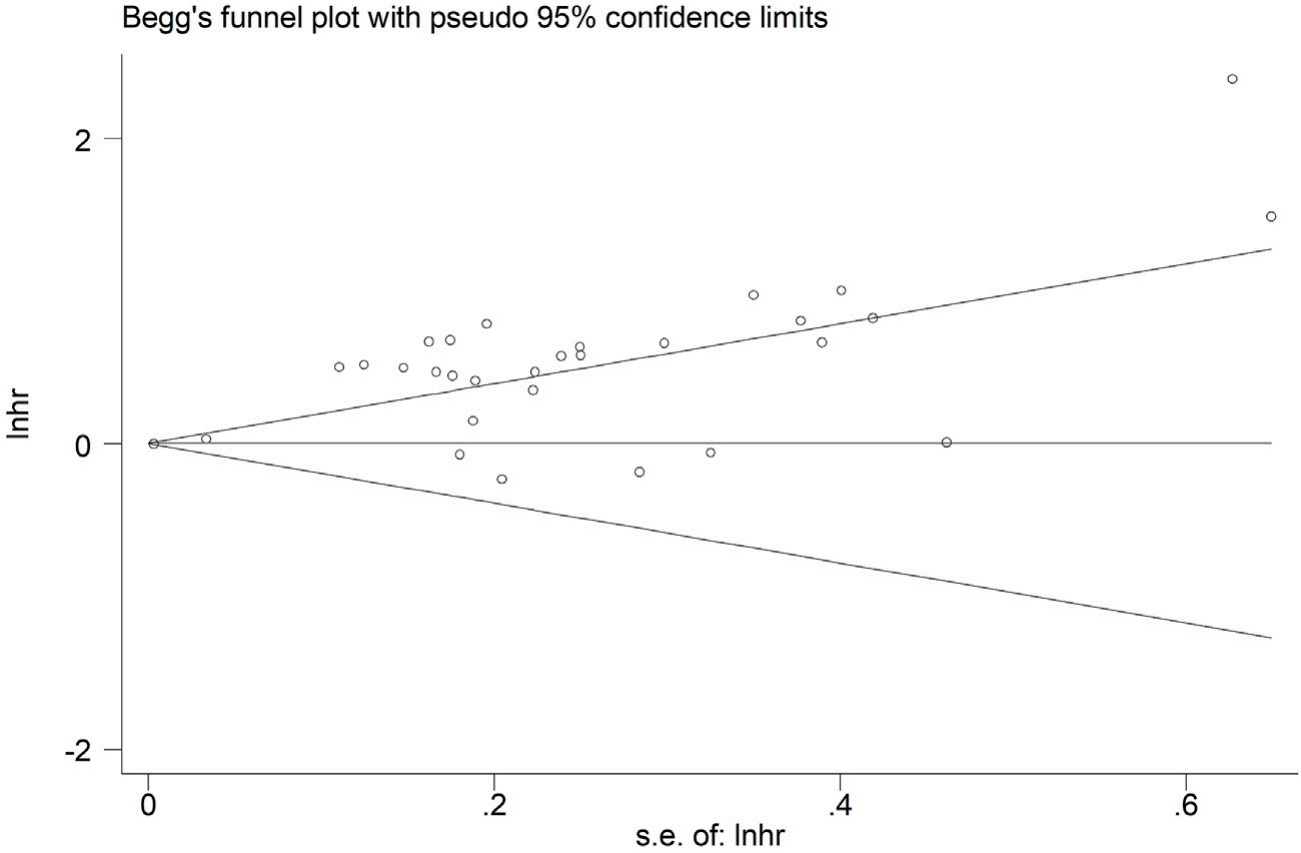
Begg’s funnel plot.

**FIGURE 6:**
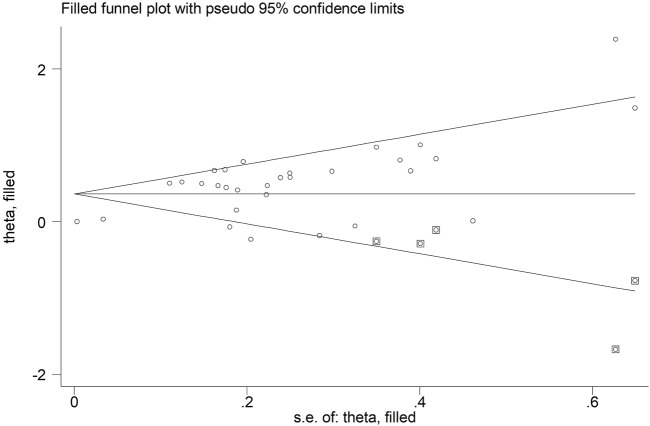
Filled Begg’s funnel plot.

Significant publication bias was observed in our meta-analysis representing as asymmetric Begg’s funnel plot (Figure 5) and *p* < 0.001 in Egger’s test. Therefore, the trim-and-fill method was further applied. As a result, there were five studies hypothetically remained unpublished for the association between pretreatment SMI and OS (Figure 6). The recalculated result did not change significantly (HR = 1.44, 95% CI: 1.28–1.61, *p* < 0.001), which indicated the stability of the results.

## Discussion

The current meta-analysis demonstrated that pretreatment SMI was significantly associated with OS, DFS and CSS of gastric cancer patients and patients with a lower pretreatment SMI suffered from poorer prognosis after reviewing 31 retrospective studies involving 12,434 patients. Thus, the pretreatment SMI should be considered during the prediction of long-term survival and formulation of appropriate treatment strategies. However, more prospective high-quality studies are still needed to verify above findings.

The SMI is usually applied to define the sarcopenia and a lot of previous literature have showed that SMI is one of the most authoritative indicators to determine the existence of sarcopenia (14, 50, 51). Sarcopenia is a condition characterized by loss of muscle mass and strength, typically associated with aging. Cachexia, on the other hand, is a multifactorial syndrome characterized by loss of muscle mass, weight loss, and systemic inflammation, often seen in chronic diseases such as cancer, heart failure, and AIDS. While both conditions involve loss of muscle mass, cachexia is a more severe and systemic condition that can cause significant morbidity and mortality. In cancer patients, sarcopenia is relatively common compared with cachexia (52). Furthermore, it has been reported that sarcopenia is frequently seen among gastric cancer patients with the incidence of 7%–57.4% in gastric cancer patients (53). Meanwhile, sarcopenia is closely related with worse prognosis of cancer patients (54–56). There are several possible causes to explain why sarcopenia worsens the survival of cancer patients. First, muscle wasting leads to increased inflammatory response (57, 58). Muscle tissue is the largest protein reservoir in the body, and when muscle wasting occurs, a large amount of protein and amino acids are released. These proteins and amino acids enter the bloodstream and stimulate an increase in the inflammatory response, which can promote tumor growth and metastasis (57, 58). Second, muscle wasting leads to decreased nutrient uptake and metabolic capacity (59). Muscle tissue has high energy and nutrient demands, and when muscle wasting occurs, the body’s metabolic rate and energy expenditure also decrease. Moreover, muscle tissue can break down protein to produce amino acids, which are essential building blocks for protein synthesis and cellular function. Muscle wasting can therefore result in amino acid deficiency, which can further affect tumor cell growth and division (59, 60). Third, muscle wasting leads to decreased immune function (61). Muscle tissue is a major reservoir for immune cells, and when muscle wasting occurs, the body’s immune function also decreases. Muscle wasting can also cause immune cells to migrate and impair their function, which reduces the body’s ability to monitor and clear cancer cells (61). Fourth, muscle wasting leads to decreased physical activity (62, 63). Muscle tissue is the primary organ for movement in the body, and when muscle wasting occurs, physical activity also decreases. Muscle wasting can also result in decreased cardiovascular and pulmonary function and reduced endurance, which can lower tolerance for cancer treatment (62, 63). Five, skeletal muscle plays an important role in enduring the chemotherapy and the chemotherapy could cause a significantly increased risk for sarcopenia (64). Thus, cancer patients with sarcopenia show a lower tolerance to chemotherapy and may experience worse therapeutic effects (65). Besides, postoperative skeletal muscle mass loss is also related to more chemotherapy modifications like the dose reduction, delay or termination and has been identified as a risk factor for worse prognosis after gastric cancer surgery, which leads to poorer tumor control and worse survival (48). Furthermore, several studies have indicated that sarcopenia is an independent predictor for worse survival of gastric cancer patients and patient with sarcopenia should be treated more positively (66–69).

Measuring and monitoring a patient’s SMI can help physicians better evaluate a gastric cancer patient’s physical condition and treatment response, and detect and intervene early in cases of malnutrition and muscle rehabilitation issues. In addition, for patients with low SMI, interventions such as reasonable nutritional support and muscle rehabilitation training can improve patients’ quality of life and treatment outcomes. Therefore, SMI can serve as an important indicator for the clinical treatment of gastric cancer patients, guiding and optimizing patients’ treatment plans and nutritional support strategies.

Actually, there are still some valuable fields about the SMI in gastric cancer needing more in-depth investigations. As mentioned above, low SMI is closely related to the adverse events of chemotherapy. Thus, we speculated that the change of SMI during the adjuvant or neoadjuvant chemoradiotherapy may also play an important role in the prediction of long-term survival of gastric cancer patients. Most of relevant studies specified the cutoff values of SMI by sex. However, age is also believed to be an essential factor that significantly affect the baseline condition of skeletal muscle mass. Therefore, more detailed and distinguishing optimal critical values of SMI are wanted, which might contribute to the clinical application of SMI. Besides, a combination of SMI and other valuable prognostic factors like the TNM stage may show higher prognostic value than a single indicator.

There are several limitations in this meta-analysis. First, all included studies are retrospective and the sample size is relatively small. Second, most studies are from Asian countries, which may limit the generalizability of the conclusions. Third, the cut off values of pretreatment SMI differ in included studies, but we could not determine the optimal critical value in this meta-analysis. Fourth, we were unable to conduct more subgroup analysis based on other important parameters such as the age, sex, and TNM stage due to the lack of original data.

## Conclusion

Pretreatment SMI was significantly related with prognosis of gastric cancer patients and lower pretreatment SMI predicted much worse survival. However, more prospective high-quality studies are still needed to verify above findings.

## Data Availability

The original contributions presented in the study are included in the article/supplementary material, further inquiries can be directed to the corresponding authors.
